# Supplementary figures and images for: De novo genome and transcriptome analyses provide insights into the biology of the trematode human parasite Fasciolopsis buski
Source: PLoS One. 2018 Oct 16;13(10):e0205570. doi: 10.1371/journal.pone.0205570 (PMC6191129; doi:10.1371/journal.pone.0205570)

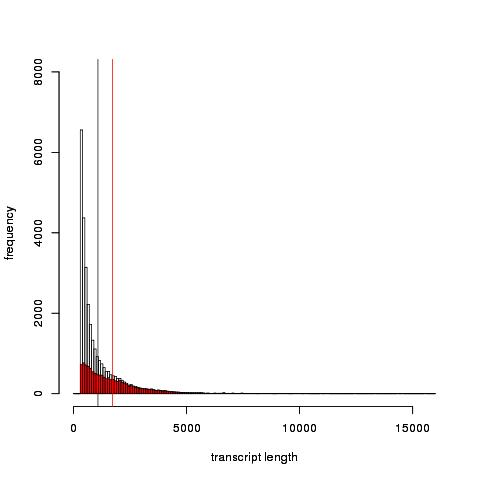

Supplement: S1 Fig — Black bars represent all unigenes while red ones represent annotated unigenes only. Clearly, mean of length of annotated unigenes is higher than overall mean length of all unigenes. (TIF) [file pone.0205570.s001.tif]
